# Prevalence and determinants of late time to treatment initiation among colorectal cancer patients in the Amhara Region, Ethiopia

**DOI:** 10.11604/pamj.2026.53.20.49889

**Published:** 2026-01-16

**Authors:** Getachew Tesfaw Walle, Seteamlak Adane Masresha, Tegene Atamenta Kitaw, Betelihem Walelign Dagnaw, Biruk Beletew Abate

**Affiliations:** 1 School of Nursing, College of Health Science, Woldia University, Woldia, Ethiopia,; 2 School of Public Health, College of Health Science, Woldia University, Woldia, Ethiopia

**Keywords:** Colorectal cancer, treatment delay, determinants, Ethiopia, oncology centers

## Abstract

**Introduction:**

timely initiation of colorectal cancer (CRC) treatment is essential for optimal patient outcomes. However, delays remain a major challenge in Ethiopia. This study aimed to assess the prevalence and determinants of late time to treatment initiation among CRC patients in oncology centers of the Amhara Region, Ethiopia.

**Methods:**

a multicenter cross-sectional study was conducted from March 30 to April 20, 2024, at Dessie, Gondar, and Felege Hiwot Oncology Centers. Data were extracted from the medical records of CRC patients diagnosed between July 1, 2018, and June 30, 2023. Bivariable and multivariable logistic regression analyses were performed. A p-value <0.05 was considered statistically significant.

**Results:**

among 464 CRC patients, 260 (56.0%; 95% CI: 51.4-60.0%) experienced delayed treatment initiation (>60 days after diagnosis). Distance from the treatment center (>81 km; AOR=3.54, 95% CI: 2.23-5.60), lack of health insurance (AOR=1.83, 95% CI: 1.20-2.78), good performance status (AOR=2.55, 95% CI: 1.53-4.24), and non-metastatic disease (AOR=1.67, 95% CI: 1.08-2.58) were significantly associated with treatment delay.

**Conclusion:**

late treatment initiation is common among CRC patients in the Amhara region. Geographic distance, lack of insurance, and certain clinical factors contribute to this delay. Efforts to decentralize oncology care and strengthen financial protection are essential to improve timely access to treatment.

## Introduction

Colorectal cancer (CRC) has emerged as a growing public health concern in Ethiopia, mirroring a wider epidemiological transition in many low- and middle-income countries (LMICs), including those in sub-Saharan Africa. This trend is driven by rapid urbanization, lifestyle shifts such as unhealthy dietary habits and physical inactivity, and improvements in life expectancy, all contributing to an increasing CRC burden [1-3]. In 2020, there were an estimated 3,121 new CRC cases in Ethiopia, making it the third most common cancer nationally, and resulting in significant morbidity and mortality [4].

Early initiation of cancer treatment is a cornerstone of optimal CRC care, yet in Ethiopia, delayed access to treatment remains a critical challenge. Historically, patients with CRC in Ethiopia have presented at advanced stages, two-thirds being stage III or IV at diagnosis, which significantly diminishes survival prospects [5]. Factors such as anemia, poor performance status, advanced tumor stage, and unfavourable tumor location have been identified as predictors of mortality among CRC patients in the Amhara Region [4]. While these insights underscore the clinical implications of late presentation, there remains a pressing need to investigate the root causes of delayed treatment initiation.

Globally, delays in CRC treatment have been linked to various sociodemographic and health system factors. A study assessing patient and provider delay in primary care, for instance, found that symptom-driven delays, such as rectal bleeding and weight loss, significantly impacted time to diagnosis and treatment initiation, even though these delays did not always correlate with advanced disease stage [5].

**Objective:** to assess the prevalence and determinants of late time to treatment initiation among colorectal cancer patients in oncology centers of the Amhara Region, Ethiopia.

## Methods

**Study design and setting:** a hospital-based cross-sectional study was conducted from March 30 to April 20, 2024, in three oncology centers of the Amhara Region: Felege Hiwot, University of Gondar, and Dessie Specialized Hospitals.

**Participants:** the study population included all CRC patients with complete follow-up data between July 1, 2018, and June 30, 2023. Patients lacking a confirmed diagnosis or missing treatment initiation dates were excluded.

**Bias and study size:** potential bias was minimized through standardized data abstraction and training of data collectors. Sample size (n=484) was determined using STATA 14 based on anticipated determinants of treatment delay.

**Variables:** the dependent variable was late time to treatment initiation (>60 days). Independent variables included sociodemographic, clinical, pathological, and treatment-related factors.

**Operational definitions/terms:** time to treatment initiation: time from the first confirmed diagnosis date of colorectal cancer to treatment initiation [6]. Considering the interval greater than 60 days as late [7]. Stage at diagnosis: stages I and II were grouped as “early stage” category, while stages III and IV were grouped as “late stage” [8].

Distance from treatment center ≤50 miles and >50 miles or ≤80km and >80km [9]. The body surface area (BSA) is ≤1.79 m^2^ and >1.79 m^2^ [10].

**Quantitative variables and statistical methods:** data were coded and entered into Epidata version 4.2, then exported to STATA version 14 for analysis. Bivariable and multivariable logistic regression analyses were performed to assess the association between independent variables and the dependent variable. Variables with a p-value <0.25 in the bivariable analysis were entered into the multivariable logistic regression model. An adjusted odds ratio (AOR) with p <0.05 and a 95% confidence interval (CI) was considered statistically significant. The model´s goodness-of-fit was evaluated using the Hosmer-Lemeshow test, which yielded a p-value of 0.2167.

**Ethical considerations:** this retrospective cohort study (July 1, 2018 - June 30, 2023) was reviewed and approved by the Institutional Review Board of Woldia University (approval reference number: WDU/IRB002). Confidentiality was maintained, and no identifiers were used. As the study used anonymized, routinely collected clinical data, the ethics committee waived the requirement for written informed consent to participate. All data were de-identified prior to analysis and handled in accordance with relevant guidelines and regulations to ensure participant confidentiality.

## Results

**Sociodemographic characteristics of participants:** a total of 464 CRC patients were included, with 96% data completeness. The mean age was 48 years (SD ±7). Most participants were male (58.6%) and urban residents (54.7%). Nearly 43% lacked health insurance coverage. Approximately forty-three percent of the participants were not covered by health insurance (Table 1).

**Table 1:** sociodemographic characteristics of late treatment initiation among colorectal cancer patients in oncology units of Amhara Region, Ethiopia, 2024 (N=464)

Characteristics	Categories	Frequency	Percent
Age	<45	211	45.47
	45-60	144	31.03
	>60	109	23.49
Sex	Male	272	58.62
	Female	192	41.38
Marital status	Married	373	80.39
	Single	49	10.56
	Others**	42	9.05
Religion	Orthodox	347	74.78
	Muslim	103	22.20
	Others**	14	3.02
Educational level	Unable to read and write	200	43.10
	Able to read and write	99	21.34
	Primary and above	165	35.56
Residence	Urban	254	54.74
	Rural	210	45.26
Health insurance	Yes	267	57.54
	No	197	42.46
Distance treatment	<80 Km	270	58.19
	>81 Km	194	41.81

*** : religion others protestants and catholics; **: marital satus others divorced and widowed

**Pathological and laboratory characteristics of participants:** more than 62% of individuals had Carcinoembryonic Antigen (CEA) levels more than 5ng/ml. Similarly, around two-fifths of participants had at least one co-morbid illness. The majority (78.9%) of histological types had adenocarcinoma (Table 2).

**Table 2:** pathological and laboratory characteristics of late treatment initiation among colorectal cancer patients in oncology units of Amhara Region, Ethiopia, 2024 (N=464)

Characteristics	Categories	Frequency	Percent
CEA level	<6 ng/ml	175	37.72
	≥6 ng/ml	289	62.28
Baseline HGB	≤12.50 gm/dl	357	76.94
	≥12.51 gm/dl	107	23.06
Histological type	Adenocarcinoma	369	79.53
	Mucinous or signet-ring cell carcinoma	95	20.47
Histological grading	Well-differentiated	173	37.28
	Moderately differentiated	122	26.29
	Poorly differentiated	85	18.32
	Undifferentiated	84	18.10
Comorbidity	Non	161	34.70
	Only one	190	40.95
	More than 2	113	24.35
Blood group	A	111	23.92
	B	95	20.47
	AB	75	16.16
	O	183	39.44
RH	RH positive	319	68.75
	RH negative	145	31.25
Baseline alkaline phosphate level	≤126 IU/l	324	69.83
	≥126.1 IU/l	140	30.17

HGB: hemoglobin; RH: rhesus; CEA: carcinoembryonic antigen

**Clinical and anthropometric characteristics of participants:** among all participants, 94.6% had a baseline body surface area (BSA) of less than 1.78m^2^. Rectal bleeding was the main presenting symptom for 70.5% of participants. Approximately 62.0% of colorectal cancer patients were diagnosed at stage III or IV (Table 3).

**Table 3:** clinical and anthropometric characteristics of late treatment initiation among colorectal cancer patients in oncology units of Amhara Region, Ethiopia, 2024 (N=464)

Characteristics	Categories	Frequency	Percent
Body surface area	≤1.78 m^2^	439	94.61
	≥1.79 m^2^	25	5.39
Presenting symptoms	Less than 2 symptoms	326	70.26
	More than 2 symptoms	138	29.74
Performance status	Poor	335	72.20
	Good	129	27.80
History of recurrent colorectal infection	Yes	277	59.70
	No	187	40.30
Tumor location	Colon	188	40.52
	Rectal	163	35.13
	Colorectal	113	24.35
Stage at diagnosis	Early	177	38.15
	Late	287	61.85

**Treatment and behavioral characteristics of participants:** among the 464 colorectal cancer patients included in the study, the most common treatment modality was chemotherapy alone, received by 308 (66.38%). Surgery combined with chemotherapy was provided to 95 (20.47%), while 50 (10.78%) underwent surgery only. A small proportion, 11 (2.37%), did not receive any form of intervention. Regarding chemotherapy regimens, the FOLFOX protocol was the most frequently administered, used in 122 (26.29%). CAPOX was given to 103 (22.20%) and FOLFIRI to 66 (14.22%). Other chemotherapy regimens were used in 32 (6.90%). Notably, 141 (30.39%) did not receive chemotherapy. In terms of lifestyle factors, 71 (15.30%) reported alcohol consumption, while the majority, 393 (84.70%), did not. Smoking was uncommon, with only 17 (3.66%) reporting a smoking history, compared to 447 (96.34%) who had never smoked.

**Prevalence of late time to treatment initiation outcome data:** of the 464 colorectal cancer patients included in the study, 56.0% (95% CI: 51.4-60.0%) of patients initiated treatment later than 60 days post-diagnosis.

**Determinants associated with time to treatment initiation:** a logistic regression analysis was performed to determine factors influencing late TTI. In the bivariable analysis, WHO or ECOG poor performance statuses, residence, health insurance, baseline body surface area, distance to treatment center, presenting symptoms, age, and metastasis showed significant associations with the outcome variable (p-value <0.25). Multivariable analysis revealed that patients residing more than 81 km from a treatment center had 3.54 times higher odds of delayed treatment initiation (AOR = 3.54, 95% CI: 2.23-5.60). Those with good WHO performance status were 2.55 times more likely to start treatment late (AOR = 2.55, 95% CI: 1.53-4.24). Rural residence was associated with a lower likelihood of delay (AOR = 0.56, 95% CI: 0.36-0.87). Similarly, having more than two presenting symptoms was associated with reduced odds of late treatment initiation (AOR = 0.53, 95% CI: 0.32-0.85). Patients without health insurance had increased odds of delay (AOR = 1.83, 95% CI: 1.20-2.78), and those without metastasis were also more likely to experience treatment delays (AOR = 1.67, 95% CI: 1.08-2.58) (Table 4).

**Table 4:** bivariable and multivariable logistic regression analysis determinants of late treatment initiation among colorectal cancer patients in oncology units of Amhara Region, Ethiopia, 2024 (N=464)

Variables	Category	Treatment initiation		COR (95% CI)	AOR (95% CI)
		**Early**	**Late**		
Age	Early age	85	126	1.56(0.98-2.49)**	1.34(0.80-2.23)
	Middle age	63	81	1.35(0.82-2.28)*	1.38(0.79-2.39)
	Older age	56	53		Base
Distance treatment	<80 Km	151	119		Base
	>81 Km	53	141	3.37(2.27-5.01)**	3.54(2.23-5.60)**
WHO performance status	Good	139	196	1.43(0.95-2.15)**	2.55(1.53-4.24)**
	Poor	65	64		Base
Residence	Urban	133	121	2.15(1.47-3.12)**	0.56(0.36-0.87)*
	Rural	71	139		Base
Presenting symptom	<2 symptoms	158	168	1.88(1.24-2.84)**	0.53(0.32-0.85)**
	>2 symptoms	46	92		Base
Health insurance	Yes	127	140		Base
	No	77	120	1.41(0.92-2.05)*	1.83(1.20-2.78)**
Baseline body surface area	≤1.78 m^2^	189	250		Base
	≥1.79 m^2^	15	10	0.50(0.22-1.21)*	0.48(0.19-1.18)
Metastasis	Yes	134	148		Base
	No	70	112	1.44 (0.99-2.01)*	1.67(1.08-2.58)*

** /*: significant variables P value <0.001 and >0.001

## Discussion

This study was reviewed at a late time to treatment initiation, and determinants of mortality among CRC patients in Oncology centers of Amhara Region registered from July 1, 2018, to June 30, 2023. A total of 464 colorectal patients were observed. In this multicenter cohort from the Amhara Region, the prevalence of late time to treatment initiation (TTI >60 days) was 56.0% (95% CI: 51.4%-60.0%). This figure is consistent with a growing body of evidence from Ethiopia and sub-Saharan Africa showing that a large proportion of patients experience substantial delays in the cancer care pathway. Several Ethiopian studies have reported similarly high magnitudes of delay in cancer care [11]. Studies focused on colorectal cancer and other solid tumours in Ethiopia also document prolonged pre-treatment and treatment intervals and advanced-stage presentation, which together likely contribute to the high TTI observed in our cohort [12].

In this study, the median time to treatment initiation was 117 days (95% CI: 95, 150). This study was higher than the study conducted in Poland (38 days) [13], Italy (28 days) [14], America (26 days) [15], 34 days [16], 68 days [17]. This discrepancy could be due to differences in healthcare infrastructure, access to healthcare services, treatment modalities, health awareness, and socioeconomic factors among the study populations.

In this study, several patient-, disease-, and system-level factors were independently associated with late treatment initiation among colorectal cancer (CRC) patients attending oncology units in the Amhara Region. The most prominent determinant was geographic distance, with patients residing more than 81 km from the treatment center having over threefold higher odds of delayed initiation compared with those living closer. This finding aligns with previous studies in Ethiopia and other low- and middle-income countries (LMICs), where travel distance remains a critical barrier due to transportation costs, prolonged travel time, and the logistical burden of repeated visits for diagnostic work-up and treatment initiation [18,19]. Decentralization of oncology services, provision of transport subsidies, and clustering of diagnostic procedures within fewer visits have been recommended to mitigate these barriers.

Residence in rural areas was also associated with delayed treatment initiation, consistent with earlier reports from Ethiopia [11,12]. Rural patients often face compounded challenges, including limited access to health information, weaker referral linkages, and lower socioeconomic resources. In contrast, urban residence likely facilitates faster care through closer proximity to specialized facilities and greater health literacy.

Financial protection emerged as a significant factor, with uninsured patients having nearly twice the odds of delayed initiation. This result is in line with international evidence demonstrating that lack of insurance or financial coverage is associated with delays in diagnosis and treatment, suboptimal care, and poorer outcomes [20]. Given the high out-of-pocket expenditure for cancer care in Ethiopia, expanding health insurance coverage and streamlining administrative processes for oncology services are crucial interventions.

Disease-related factors also influenced time to treatment. Patients presenting with more than two symptoms were more likely to experience delays, potentially reflecting more advanced or complex disease requiring extensive staging, multidisciplinary input, and pre-treatment optimization. Similar associations have been reported in other oncology cohorts, where higher symptom burden is linked to longer pre-treatment intervals [21]. Moreover, patients without metastases were more likely to have delayed initiation compared to those with metastatic disease. This paradoxical finding may be explained by clinical prioritization: advanced cases are often fast-tracked for systemic therapy, while non-metastatic patients undergo additional preoperative preparation and scheduling, potentially prolonging the initiation interval [22]. An unexpected but noteworthy finding was that patients with good WHO performance status had a higher likelihood of delayed initiation. While counterintuitive, this may reflect triage practices that prioritize patients with poorer performance status due to urgent clinical need, or patient-driven delays among those who feel relatively well [23]. This highlights the importance of establishing maximum acceptable wait times for all patients, regardless of apparent urgency.

Variables such as age and body surface area did not demonstrate significant associations in the adjusted analysis, suggesting that, in this setting, structural and socioeconomic barriers outweigh demographic factors in determining timeliness of care. The findings of this study underscore the need for multi-pronged strategies to reduce treatment delays. These include decentralization of oncology services to zonal hospitals, targeted financial support for uninsured and rural patients, implementation of patient navigation programs, and streamlined diagnostic and surgical pathways for non-metastatic CRC cases. Reducing delays is particularly critical given evidence linking longer time-to-treatment intervals with poorer survival outcomes in CRC [24].

**Limitations:** the cross-sectional nature limits causal inference, and performance status or symptom burden may reflect disease severity rather than act as direct causes of delay. Despite these limitations, the large sample size and adjustment for multiple confounders strengthen the validity of the observed associations.

## Conclusion

Late treatment initiation among CRC patients is common in the Amhara Region and is driven by a combination of geographic, financial, and system-level factors. Policy actions should focus on decentralizing oncology services and expanding insurance coverage to improve timely access.

### What is known about this topic

- Delayed cancer treatment initiation contributes to poor survival outcomes in low-resource settings;
- Geographic and socioeconomic barriers are major determinants of cancer treatment delay in sub-Saharan Africa;
- Evidence on colorectal cancer treatment delays in Ethiopia is limited.

### What this study adds

- This study provides the first multicenter analysis of colorectal cancer treatment delay in the Amhara Region;
- It identifies geographic distance (>81 km) and lack of insurance as key determinants of delayed initiation;
- And also highlights paradoxical findings where patients with good performance status and non-metastatic disease experience longer delays.
